# Investigating the Role of Telomere and Telomerase Associated Genes and Proteins in Endometrial Cancer

**DOI:** 10.3390/mps3030063

**Published:** 2020-09-03

**Authors:** Alice Bradfield, Lucy Button, Josephine Drury, Daniel C. Green, Christopher J. Hill, Dharani K. Hapangama

**Affiliations:** 1 Department of Women’s and Children’s Health, University of Liverpool, Crown St, Liverpool L69 7ZX, UK; a.j.bradfield@liverpool.ac.uk (A.B.); jadrury@liverpool.ac.uk (J.D.); C.J.Hill1@liv.ac.uk (C.J.H.); 2 Faculty of Health and Life Sciences, University of Liverpool, Brownlow Hill, Liverpool L69 7ZX, UK; Lucy.Button@liverpool.ac.uk; 3 Institute of Life Course and Medical Sciences, Faculty of Health and Life Sciences, University of Liverpool, Liverpool L7 8TX, UK; Daniel.Green@liverpool.ac.uk; 4 Liverpool Women’s NHS Foundation Trust, Member of Liverpool Health Partners, Liverpool L8 7SS, UK

**Keywords:** telomere, telomerase, endometrial cancer, prognosis, bioinformatics analysis, transcriptome, TCGA

## Abstract

Endometrial cancer (EC) is the commonest gynaecological malignancy. Current prognostic markers are inadequate to accurately predict patient survival, necessitating novel prognostic markers, to improve treatment strategies. Telomerase has a unique role within the endometrium, whilst aberrant telomerase activity is a hallmark of many cancers. The aim of the current in silico study is to investigate the role of telomere and telomerase associated genes and proteins (TTAGPs) in EC to identify potential prognostic markers and therapeutic targets. Analysis of RNA-seq data from The Cancer Genome Atlas identified differentially expressed genes (DEGs) in EC (568 TTAGPs out of 3467) and ascertained DEGs associated with histological subtypes, higher grade endometrioid tumours and late stage EC. Functional analysis demonstrated that DEGs were predominantly involved in cell cycle regulation, while the survival analysis identified 69 DEGs associated with prognosis. The protein-protein interaction network constructed facilitated the identification of hub genes, enriched transcription factor binding sites and drugs that may target the network. Thus, our in silico methods distinguished many critical genes associated with telomere maintenance that were previously unknown to contribute to EC carcinogenesis and prognosis, including *NOP56*, *WFS1*, *ANAPC4* and *TUBB4A*. Probing the prognostic and therapeutic utility of these novel TTAGP markers will form an exciting basis for future research.

## 1. Introduction

Endometrial cancer (EC) is the most common gynaecological cancer and fourth most common cancer in women in the UK [1]. Overall, EC has a good prognosis with 78% of patients achieving 10-year survival [1]. Currently, our only methods of determining which patients are more likely to suffer poor outcomes include clinicopathological features such as tumour grade, histological subtype and clinical stage [2]. Hysterectomy with or without adjuvant radiotherapy is curative for most patients. However, a small subset of patients will develop a disease recurrence that fails to respond to chemotherapy and thus experience shorter survival [2]. This group has proven difficult to identify at diagnosis, therefore a novel prognostic marker may be of particular benefit for these patients. With the rising incidence of EC and associated mortality [3], better provision of care will be essential in the future, further reinforcing the need for novel prognostic markers.

Historically, EC has been categorised into type I and type II cancers. Type I comprises 80% of EC diagnoses and consists of early grade, early stage tumours that are of the endometrioid subtype and are often oestrogen-responsive with a low rate of recurrence [4]. Type II cancers are high grade, have a high frequency of metastasis and are associated with poorer patient outcome [5]. Despite comprising only 20% of cases, type II cancers are responsible for 40% of EC-related deaths [4]. Type II EC includes grade 3 endometrioid and all other histological subtypes, including serous, clear cell, carcinosarcoma, squamous, mucinous, neuroendocrine and undifferentiated [6].

**Figure 1:**
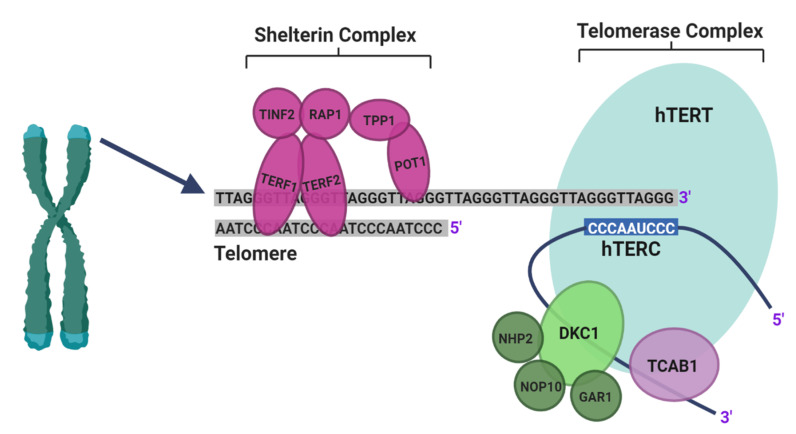
Schematic illustration of telomeres and the main components of telomerase, adapted from Hapangama et al. [12]. Telomerase is a holoenzyme comprising three core components: human telomerase reverse transcriptase (hTERT), human telomeric RNA component (hTERC) and dyskerin (DKC1). hTERT is a catalytic protein with transcriptase activity and hTERC provides the RNA template from which new telomeric DNA is synthesized [12]. NHP2, NOP10 and GAR1, in addition to DKC1, bind the H/ACA snoRNA motif at the 3′ end of hTERC and stabilise newly transcribed telomeric RNA. The H/ACA region also binds telomerase Cajal body protein 1 (TCAB1). The shelterin complex is made up of telomeric repeat binding factors 1 and 2 (TERF1 and TERF2), repressor/activator protein 1 (RAP1), protection of telomeres 1 (POT1), TERF1 interacting nuclear factor 2 (TINF2) and TPP1 (encoded by the gene *ACD*). POT1 binds directly to the single stranded 3′ end of the telomere and forms a heterodimer with TPP1. TERF1 and TERF2 bind to the double-stranded telomeric sequence [11]. (Created with BioRender.com).

Telomeres are specialised structures that protect the ends of chromosomes and help to maintain genomic stability [7]. In addition to this, they limit cellular proliferation by shortening in length with each round of DNA replication until they reach a critical length, which induces permanent cell-cycle arrest [8,9,10]. Telomere length can be regulated by one of two mechanisms: the well-established telomerase dependent pathway or by the more recently described alternative lengthening of telomeres (ALT) pathway [11]. Telomerase is a reverse transcriptase enzyme that synthesizes telomeric DNA sequences using an RNA template (Figure 1) [7]. In contrast, the ALT pathway utilises homologous recombination repair to synthesise new telomeric DNA [11].

The unlimited proliferative capacity of cancer cells can, in part, be attributed to aberrant telomerase activity, which is repressed in most somatic cells but present in up to 90% of cancers [12,13]. Furthermore, higher telomerase activity has been correlated with more aggressive/advanced cancers, suggesting that it may contribute to the poorer outcomes associated with some cancers [14,15]. These features make telomerase a useful therapeutic target and consequently, many telomerase-based therapies have been investigated as prospective anti-cancer treatments [16]. However, telomerase has a unique role in the benign endometrium, as this is one of the few somatic tissues to already exhibit significant telomerase activity [17,18,19]. The significant regenerative capacity of the endometrium may be the reason for this, as well as the cyclical endometrial proliferation and shedding with each menstrual cycle [12]. The endometrium expresses a dynamic pattern of telomerase activity throughout the cycle, in which levels are highest in the proliferative and lowest in the secretory phase [12,20,21,22]. Telomerase activity is also affected by steroid hormones, and it is upregulated by oestrogen and inhibited by progesterone [20]. It may be via this mechanism that progesterone administration slows tumour progression in the secondary management of EC [20,23].

Endometrial carcinogenesis is not well understood. Considering the unique role telomerase appears to play within the human endometrium, characterisation of telomere and telomerase associated genes and proteins (TTAGP) that are aberrantly expressed in EC may provide further insight into their diagnostic, prognostic and therapeutic utility. The aim of the current in silico study was therefore to investigate the role of TTAGPs in EC and identify potential prognostic markers and therapeutic targets of disease. This was undertaken with bioinformatic analysis of the RNA expression dataset for EC cohort from The Cancer Genome Atlas (TCGA) database.

## 2. Experimental Design

### 2.1. Identification of TTAGPs

**Figure 2:**
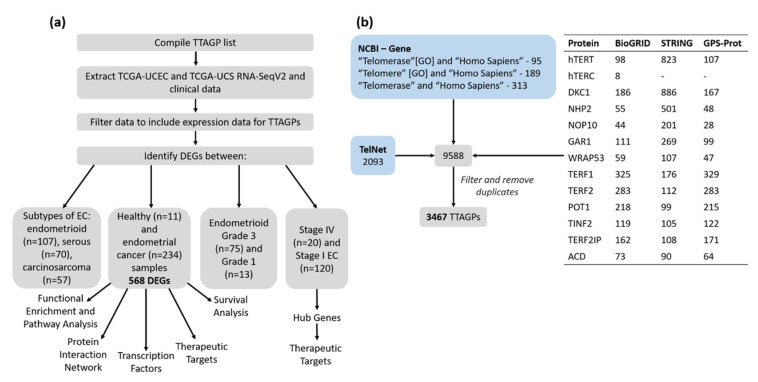
(**a**) Workflow diagram illustrating the in silico procedures. (**b**) Database searches for compiling the list of TTAGPs. The table displays the number of interactors identified for each protein within the telomerase and shelterin complex. *WRAP53* and *TERF2IP* are the official gene symbols for TCAB1 and RAP1, respectively. TPP1 is encoded by the *ACD* gene.

A diagram displaying the workflow for the current study is shown in Figure 2a. Database searches were undertaken to compile a comprehensive list of genes and proteins that associate with telomerase and are involved in telomere maintenance (Figure 2b). A total of 3467 TTAGPs were identified from five databases: TelNet, National Center for Biotechnology Information (NCBI– Gene (www.ncbi.nlm.nih.gov/gene/), Biological General Repository for Interaction Datasets (BioGRID) (https://thebiogrid.org/), Search Tool for the Retrieval of Interacting Genes/Proteins (STRING) (https://string-db.org/) and GPS-Prot (http://gpsprot.org/) [24,25,26,27,28,29,30,31]. TelNet contains over 2000 genes related to telomere maintenance and attributes a TelNet score to each gene, representing its significance to telomere maintenance (http://www.cancertelsys.org/telnet) [32]. Interactors for each component of the telomerase and shelterin complex were identified using BioGRID, STRING and GPS-Prot databases. The interaction score was set at medium confidence (≥0.400) throughout. Within the STRING database, first and second shell interactors were included for hTERT and DKC1, as these form core components of the telomerase holoenzyme, and all first shell interactors were included for the remaining proteins. Interactors for *hTERC* were excluded from STRING and GPS-Prot as it is a long non-coding RNA. Duplicates and genes that were non-human were manually removed to generate the final list.

### 2.2. TCGA Data Cohort

RNASeq and clinicopathological data for EC samples were downloaded from TCGA database (https://www.cancer.gov/tcga), using Broad Genome Data Analysis Centre (GDAC) FireHose (gdac.broadinstitute.org) (Figure 2a). A total of 234 cancer and 11 healthy patient samples had available normalised RNASeqV2 data and were included in the study. EC samples consisted of those from both the Uterine Corpus Endometrial Carcinoma (TCGA-UCEC) and Uterine Carcinosarcoma (TCGA-UCS) datasets. The interrogation of anonymous, public and freely available mRNA expression data provided by TCGA does not require ethics committee approval.

### 2.3. Identification of Differentially Expressed Genes (DEGs)

DEG analysis was performed between the following categories: cancer and healthy endometrium, histological subtypes of EC, grade 1 and 3 endometrioid tumours, and stage I and IV EC (Figure 2a). Tumours with mixed endometrioid and serous histology were categorised as serous tumours. Differential expression analysis was conducted using limma in the web application iDEP.91 (Integrated Differential Expression and Pathway analysis) (http://bioinformatics.sdstate.edu/idep/) [33]. A │log2FC > 1│ and false discovery rate (FDR) <0.01 were used as cut-off criteria for DEGs [34]. Venn diagrams were constructed using the Bioinformatics and Evolutionary Genomics web-tool (http://bioinformatics.psb.ugent.be/webtools/Venn/). Principle component analysis (PCA) was conducted using the prcomp function from the stats packages within R (https://cran.r-project.org/).

### 2.4. Functional Enrichment and Pathway Analysis

Enrichr (https://amp.pharm.mssm.edu/Enrichr/) was used for the functional analysis of DEGs [35,36]. Gene Ontology (GO) analysis was performed to elucidate the molecular functions, biological processes and cellular components associated with the DEGs. Kyoto Gene and Genome Encyclopaedia (KEGG) enrichment analysis was also performed to gain insight into the associated signalling pathways. Adjusted *p* < 0.05 was chosen as a cut-off. The web tool REVIGO (http://revigo.irb.hr/) was utilised to reduce redundancy of the GO terms and condense them into a smaller representative subset [37]. Similarity of GO terms was set at <0.5.

### 2.5. Protein–Protein Interaction (PPI) Network

A PPI network of DEGs was constructed with interaction data from STRING, and this was visualised with Cytoscape version 3.8.0 (http://www.cytoscape.org/) [28,38]. The minimum confidence score was set at 0.400 and only nodes with a degree ≥1 were included in the network. In order to identify modules within the network, the Molecular Complex Detection (MCODE) plug-in was used (degree cut-off ≥ 2) [39]. This identifies densely connected regions within a network based on topology. The Cytohubba plug-in was used to select the top 10 hub genes within the entire network, according to degree [40].

### 2.6. Identification of Key Transcription Factors (TFs)

oPOSSUM 3.0 (http://opossum.cisreg.ca/oPOSSUM3/) was used to identify over-represented transcription factor binding sites (TFBS) amongst the DEGs [41,42]. Human single site analysis was performed, in which the genes were compared against all 24,752 genes in the oPOSSUM database, using all vertebrate JASPAR CORE transcription factor profiles. Sequences were searched +/−2000 bp from the start sites of each gene. A conservation cut-off was set at 0.60 and a matrix score threshold at 80%. Results were analysed according to Fisher score. This score compares the proportion of a set of genes containing a particular TFBS motif to the proportion of the background set that contains the motif [41]. When analysed by Z-score, this showed some bias in identifying TFs with a lower GC content (Figure S1a). As a result, TFs were identified according to Fisher score that showed a more even distribution (Figure S1b). A Fisher score greater than 2 standard-deviations above the mean was used as a cut-off for selecting TFs. Due to the large number of genes included in the analysis, a control analysis was performed using 2 sets of 2000 randomly selected genes that were not differentially expressed in EC. This ensured that the results were not due to chance.

### 2.7. Therapeutic Targets

The Drug Gene Interaction Database (DGidb) was screened to identify known associated drugs for hub genes and enriched TFs [43].

### 2.8. Survival Analysis

The survival information for each DEG in EC was taken from The Human Protein Atlas (http://www.proteinatlas.org), which is based upon clinical information from all patients within the TCGA-UCEC dataset (*n* = 541) [44]. Genes that had a significant association with overall survival (*p* < 0.001, Log-rank test) were regarded as prognostic in EC. The cut off value for high and low expression differs for each gene, and is based upon the value which yields the maximal difference in survival and the lowest log-rank *p*-value.

## 3. Results

### 3.1. Identification of TTAGPs and EC-Associated DEGs

**Figure 3:**
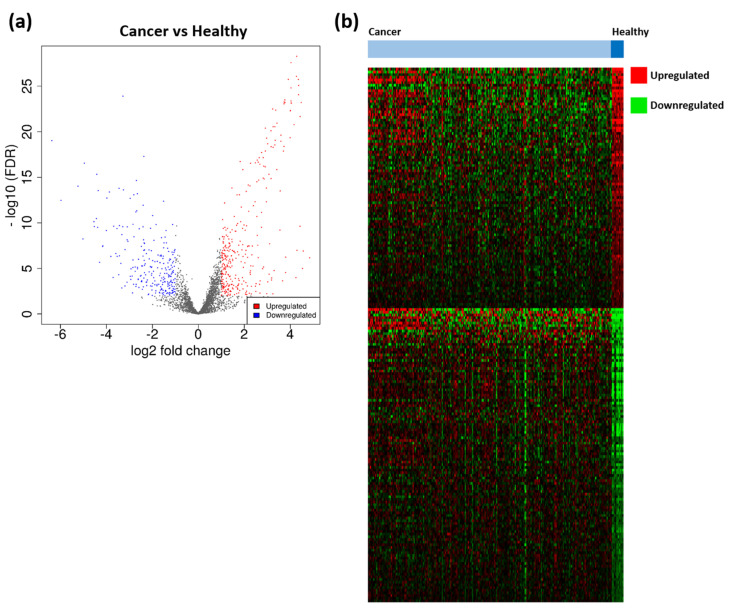
Differentially expressed genes (DEGs) identified between endometrial cancer (EC) and healthy endometrium. (**a**) Volcano plot of DEGs amongst cancer (*n* = 234) and healthy samples (*n* = 11). Significant DEGs are coloured; red dots represent upregulated genes, and blue dots represent downregulated genes. Cut-off criteria: │log2FC > 1│and false discovery rate (FDR) < 0.01. (**b**) Heatmap displaying the expression of 568 DEGs. Red denotes upregulated genes and green denotes downregulated genes.

A total of 3467 TTAGPs were identified from database searches (Table S1). Out of these, 75 genes were not found within the TCGA datasets and consequently, 3392 genes were included in DEG analysis. TCGA RNA expression data and clinical data is available in Tables S2–S4. 568 telomerase associated DEGs were identified between EC (*n* = 234) and healthy endometrium (*n* = 11). A greater number of DEGs were upregulated (323) in cancer than downregulated (245) (Figure 3). A full list of DEGs with their associated TelNet scores and ranked by log2FC is available in Table S5. Of the 568 DEGs, 192 were not listed on TelNet and therefore did not have TelNet scores. The top 5 upregulated DEGs, ranked by log2FC, included *JSRP1, IGF2BP3, FOXA1, CDC45* and *BIRC5*. The top 5 downregulated DEGs, by log2FC, were *MYOCD, RSPO1, FOXL2, WT1* and *ARHGAP20*. Additional EC-associated DEGs with high TelNet scores included *hTERT, BLM, FEN1, RUVBL1* and *HSP90AA1*, which were all upregulated.

### 3.2. DEGs Associated with Histological Subtypes of EC

**Figure 4:**
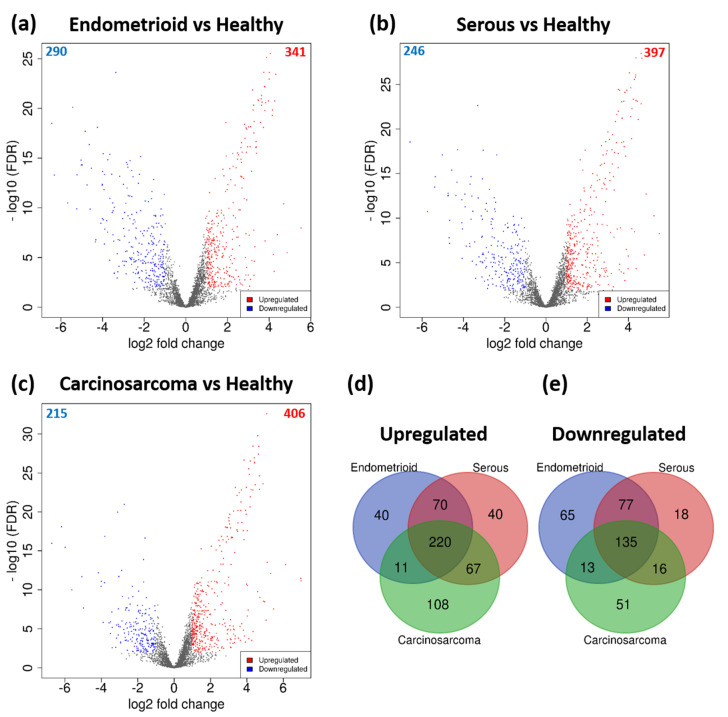
Volcano plots of DEGs between (**a**) endometrioid and healthy endometrium, (**b**) serous and healthy, and (**c**) carcinosarcoma and healthy. Significant DEGs are coloured; red dots represent upregulated genes, and blue dots represent downregulated genes. Cut-off criteria: │log2FC > 1│and FDR < 0.01. Venn diagrams displaying common (**d**) upregulated and (**e**) downregulated genes between each subtype.

A total of 631 DEGs were identified between endometrioid tumours (*n* = 107) and healthy (*n* = 11) endometrium, of which 341 were upregulated and 290 downregulated (Figure 4a, Table S6). Between serous tumours (*n* = 70) and healthy endometrium, 643 DEGs were identified. Out of which, 397 were upregulated and 246 were downregulated (Figure 4b, Table S7). There were 621 DEGs identified between carcinosarcoma (*n* = 57) and healthy endometrium, of which 406 were upregulated and 215 were downregulated (Figure 4c, Table S8). There were 220 genes consistently upregulated across all subtypes, including *TERT, FEN1, BLM, PCNA, AURKA* and *PITX1* (Figure 4d, Table S9). There were 135 genes downregulated across all subtypes that were identified, such as *KLF4, NR2F2, KLF2, EGR1, ETS2* and *AR* (Figure 4e, Table S10). There were 105 endometrioid-specific DEGs that were identified, and the highest upregulated genes included *CEACAM5, S100P* and *PCSK9*, and the highest downregulated genes were *IQSEC3, H19* and *ELOVL4*. There were 58 genes dysregulated in only the serous subtype. The highest upregulated serous-specific genes were *XAGE2, CCDC155* and *AIM2*, and the most highly downregulated were *PCP4, TBX1* and *DLG2*. There were 159 carcinosarcoma-specific DEGs identified, including the upregulated genes *MYOG, DMRT2* and *SLC7A10*, and the downregulated genes *WDR38, PHYHD1* and *POU5F1*.

Healthy controls separated from EC samples on a PCA plot of telomerase-associated transcripts and separation was determined by PC3 (Figure S2). There was also some separation of carcinosarcoma and endometrioid samples on the PCA plot and this was determined by PC2. From the PCA loading plot, we identified the top 50 genes from each principal component contributing to variance. PC3 included genes such as *ARHGAP20*, *FOXL2*, *MYOCD*, *RSPO1* and *IGF2BP3*, whilst PC2 included *MYOG*, *TUBB2B*, *CEACAM5*, *HGD* and *WDR38*.

### 3.3. DEGs Associated with Tumour Grade and Clinical Stage

**Figure 5:**
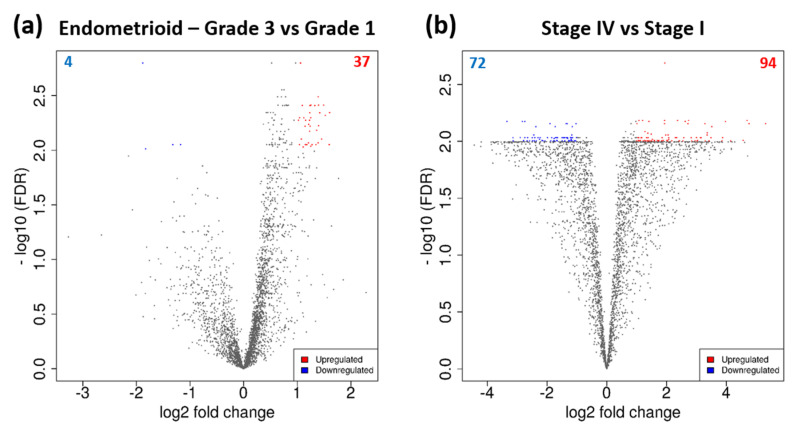
DEGs associated with tumour grade and clinical stage. Volcano plots of DEGs between (**a**) grade 1 and grade 3 endometrioid, and (**b**) stage I and IV EC. Significant DEGs are coloured; red dots represent upregulated genes, and blue dots represent downregulated genes. Cut-off criteria: │log2FC > 1│and FDR < 0.01.

Between grade 1 (*n* = 13) and grade 3 (*n* = 75) endometrioid tumours, 37 genes were upregulated and four genes were downregulated (Figure 5a, Table S11). The most highly upregulated genes in grade 3 were *CDC45*, *RAD51AP1, PKMYT1* and *KIAA0101,* whilst *IGFBP4*, *GLI1*, *HIC1* and *PTCH1* were downregulated. 166 DEGs were identified between clinical stage I (*n* = 120) and stage IV (*n* = 20) ECs, out of which 94 were upregulated and 72 were downregulated (Figure 5b, Table S12). The most highly upregulated DEGs included *MAGEA4, SULT1E1*, *TDRD10*, and *XAGE2*, and the most highly downregulated were *DUT, SETDB1*, *SRP9* and *ZNF140*.

### 3.4. Functional Enrichment and Pathway Analysis

**Figure 6:**
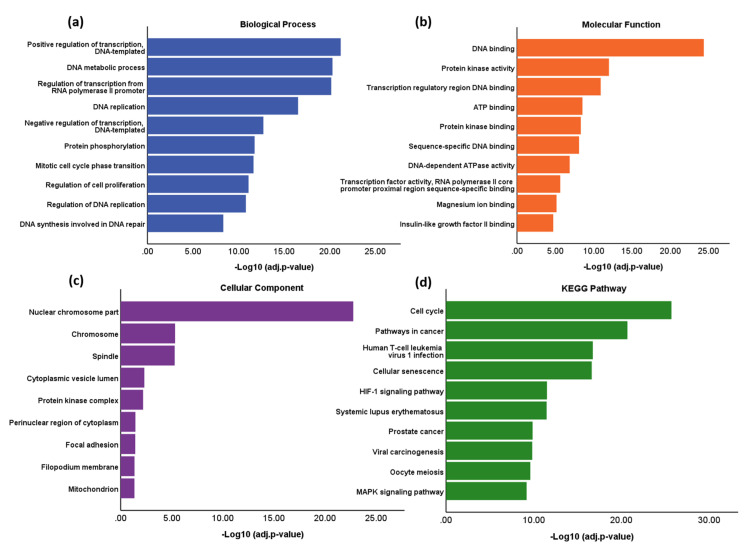
Functional Enrichment and Pathway Analysis of DEGs. GO terms and Kyoto Gene and Genome Encyclopaedia (KEGG) pathways were identified using Enrichr. The GO terms were subsequently revised into a smaller representative list using REVIGO (similarity <0.5). (**a**) Biological Process. (**b**) Molecular Function. (**c**) Cellular Component. (**d**) KEGG pathway.

GO function and KEGG pathway enrichment analysis was performed to assess the functional significance of the 568 telomere and telomerase associated DEGs. A total of 429 significant GO terms of biological process, 105 GO terms of molecular function and 44 GO terms of cellular component were identified from Enrichr. After using REVIGO, 48 biological process terms, 40 molecular function terms and nine cellular component terms remained. The full list of GO terms and KEGG pathways is presented in Tables S13–S19. Biological process terms were predominantly associated with regulation of transcription and cellular division (Figure 6a). For molecular function, DEGs showed significant enrichment in DNA binding and regulation of transcription (Figure 6b). The results amongst cellular component analysis showed that DEGs were enriched in the chromosome and spindle, suggesting a role within DNA replication (Figure 6c). There were 96 significant KEGG pathways identified and these included ‘cell cycle’ and ‘pathways in cancer’ (Figure 6d). Overall, many functional terms and pathways identified were associated with DNA replication, the cell cycle and regulation of transcription.

### 3.5. PPI Network

**Figure 7:**
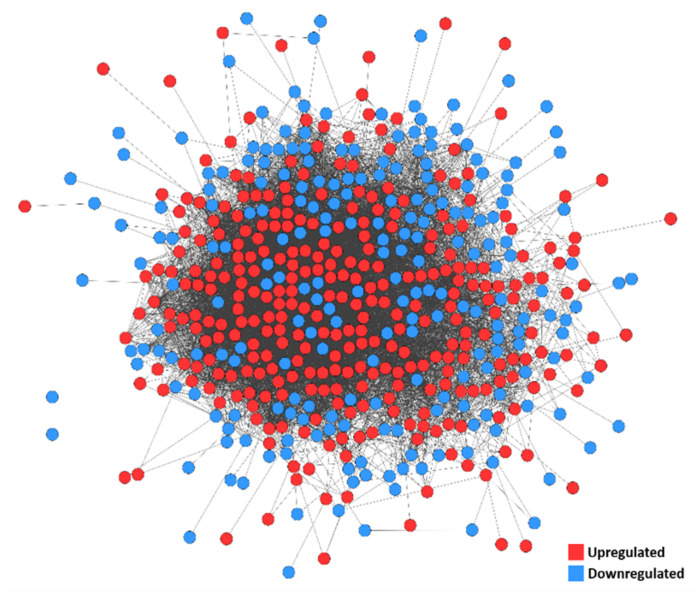
Protein–Protein Interaction (PPI) network of DEGs. Upregulated and downregulated DEGs are represented by red and blue nodes, respectively. Degree ≥ 1.

**Figure 8:**
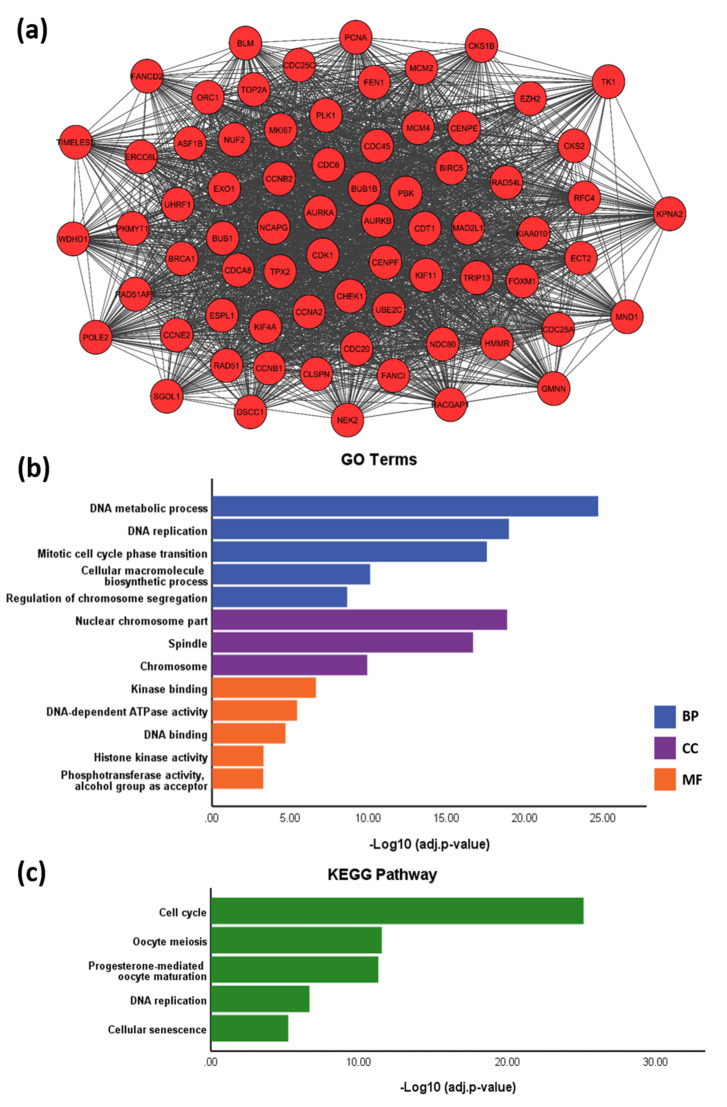
(**a**) The module identified in the PPI network of DEGs using Molecular Complex Detection (MCODE). MCODE score = 64.171. Degree cut-off ≥ 2. (**b**) GO terms and (**c**) KEGG pathways associated with the module. Abbreviations: BP—Biological Process; CC—Cellular Component; MF—Molecular Function. GO terms and KEGG pathways were identified using Enrichr (adjusted *p* < 0.05). The GO terms were subsequently summarised into a smaller representative list using REVIGO (similarity < 0.5).

The PPI network was constructed from DEGs with a degree ≥1 and consisted of 535 nodes (proteins) and 9001 edges (interactions), including 309 upregulated and 226 downregulated DEGs (Figure 7; Table S20). Using MCODE, a module with a score of 64.171 was identified (Figure 8a, Table S21). This was made up of 71 nodes and 2246 edges, and all nodes within the module were upregulated DEGs. Significant biological process GO terms for this module included ‘DNA replication’ and ‘mitotic cell cycle phase transition’ (Figure 8b, Tables S22–S28). For molecular function analysis, the module showed predominant enrichment in DNA binding. Significant cellular component GO terms included ‘nuclear chromosome part’, ‘spindle’ and ‘chromosome’. KEGG pathway analysis suggested an association with ‘cell cycle’, ‘DNA replication’ and ‘cellular senescence’ (Figure 8c). Taken together, the results suggest that this module is predominantly associated with DNA replication and cell cycle regulation.

**Figure 9:**
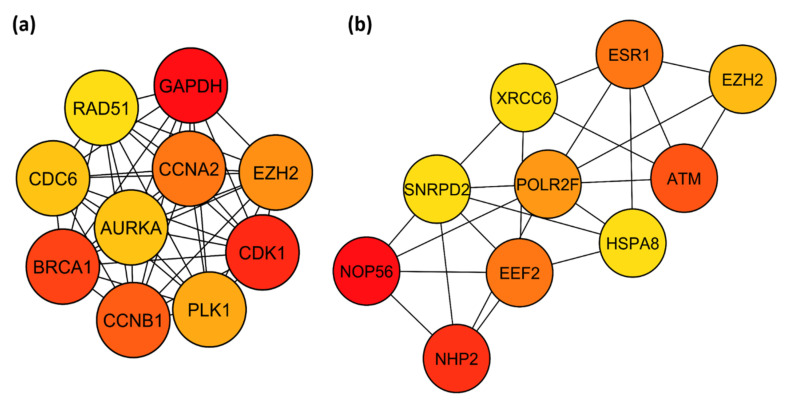
Top 10 hub genes of the PPI network constructed from (**a**) EC-specific DEGs and (**b**) stage I-IV DEGs, ranked according to degree. The hub genes were identified using Cytohubba. The colour of the node represents degree, with red representing a higher degree and yellow a lower degree.

Using cytohubba, all nodes within the network were ranked according to degree and the top 10 were selected. This included *GAPDH, CCNB1* and *CDC6* (Figure 9a, Table S20). Degree represents the number of nodes within the network that a node interacts with and thus, nodes with a higher degree may be more likely to influence the regulation of others within the network. The top 10 hub genes were then also identified from DEGs between stage I and IV EC; *NOP56* and *NHP2* had the highest degrees of 29 and 28, respectively (Figure 9b, Table S29).

### 3.6. Identification of Key TFs

From oPOSSUM analysis of DEGs, three enriched TFBS were identified: MZF1_5-13, ZEB1 and E2F1 (Table 1, Table S30). This is supported by results from the control analysis, in which none of these TFBS were above the cut-off criteria (Tables S31–S32). All three of the identified TFs have an association with telomeres and telomerase (Table S1). In addition, *ZEB1* is downregulated and *E2F1* is upregulated in EC compared to healthy endometrium (Table S5).

### 3.7. Therapeutic Targets

Using the DGidb, known drugs associated with enriched TFs and hub genes from the PPI network, in addition to hub genes from stage I–IV DEGs, were identified (Table S33). This included metformin, ibrutinib, AURKA inhibitors, cordycepin, genistein, suramin, sodium butyrate, SS1(dsFv)-PE38 and AZD-6482. Everolimus (mammalian target of rapamycin (mTOR) inhibitor) and poly (ADP-ribose) polymerase (PARP) inhibitors, such as olaparib, veliparib, talazoparib, are known to target ataxia telangiectasia mutated (ATM) and breast cancer type 1 susceptibility protein (BRCA1). Multiple chemotherapy agents target the hub genes and TFs, including carboplatin, paclitaxel, doxorubicin, chlorambucil, carmustine and bendamustine. Mitogen-activated protein kinase (MEK) inhibitors, such as selumitinib, binimetinib and trimetinib, were identified that target ATM and EZH2. Many cyclin-dependent kinase inhibitors were also identified, such as variolin B, meriolin, alsterpaullone and dinaciclib, which target CCNA2 and CDK1. No drugs were associated with CCNB1, CDC6, MZF1, NOP56, NHP2, POLR2F, XRCC6 and SNRPD2.

### 3.8. Survival Analysis

Using prognostic data from The Human Protein Atlas, 69 out of 568 EC-specific DEGs had a significant effect upon overall survival in EC (Log-rank test, *p* < 0.001) (Table S34). Twenty DEGs had a favourable effect, in which high expression was associated with longer overall survival. The most significant favourable prognostic DEGs were *ESR1, ANAPC4, RPS6KA1* and *WFS1*. There were 49 DEGs associated with an unfavourable prognosis, such as *ERBB2*, *ARL4C*, *TUBB4A, TPX2, AURKA* and *CCNA2*. This prognostic data is based only upon RNA expression data from the TCGA-UCEC dataset (*n* = 541), and thus does not include data from carcinosarcoma samples (TCGA-UCS). Out of 541 patients, a total of 91 deaths occurred. Some genes in the TTAGP list that were not dysregulated in EC compared to healthy endometrium were also found to be associated with prognosis, for example, *NOP56* [44].

**Figure 10:**
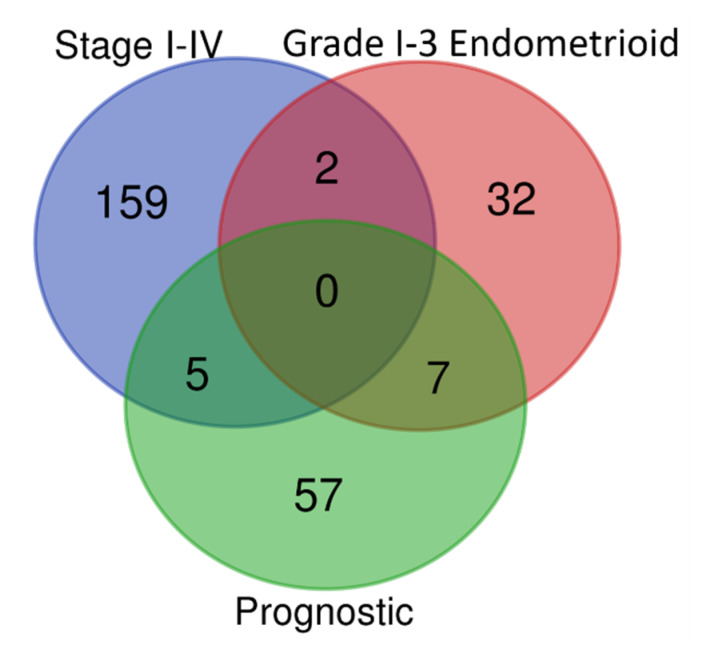
Venn diagram displaying the intersections of stage I-IV DEGs, Grades 1–3 DEGs and prognostic DEGs.

As genes dysregulated in higher grade or later stage disease may indicate that a tumour is more aggressive, the list of DEGs from the comparison of stage I and IV EC, and grade 1 and 3 endometrioid, were intersected with the list of prognostic genes (Figure 10, Table S35). There was very little overlap between the groups and no DEGs were common across all three groups. The 7 DEGs that were dysregulated in grade 3 endometrioid cancer and were also prognostic genes in EC (*TPX2*, *AURKA*, *ATAD2, IGFBP4, CKS1B, NCAPG* and *RAD51AP1*), were all upregulated and associated with poor prognosis, except *IGFBP4* that was downregulated and linked with a favourable prognosis. Five DEGs were linked with both stage IV disease and prognosis; *ESR1, CIRBP* and *GLTSCR2* were downregulated and linked with a favourable prognosis, whereas *CDKN2B* and *MRPL47* were upregulated and associated with poor prognosis. Furthermore, two genes were commonly downregulated in both stage IV disease and grade 3 endometrioid (*KIF4A* and *UBE2C*).

## 4. Discussion

Telomere maintenance is a complex, multistep process that is regulated by a large number of proteins as evidenced by our database search [32,45,46,47]. The dysregulation of many telomere maintenance genes and proteins have been linked to telomere shortening and telomerase activity in cancer [48]. Despite previous studies demonstrating that hTERT expression and telomerase activity correlate with poor survival in multiple cancers [14,49,50,51,52,53,54], this has not been seen in EC. This may be due to both hTERT expression and telomerase activity being normally active in the benign endometrium already [17,18,19,55]. In this study, by considering a wider network of TTAGPs, we have been able to identify genes and proteins that are linked, through their shared influence on telomere biology, to endometrial carcinogenesis, progression and survival.

When comparing the expression of TTAGPs between EC and healthy endometrium, *hTERT* and multiple associated genes, such as *HSP90AA1* and *RUVBL1*, were upregulated, agreeing with prior reports of an increase in telomerase activity in EC [56,57]. Our work has highlighted some novel bio-targets relevant to telomere/telomerase biology that may play a role in EC. For example, *JSRP1* was the most highly upregulated DEG. Little is known about its functions, except that it is involved in excitation–contraction coupling at the sarcoplasmic reticulum in skeletal muscle [58]. A fluorescence localisation screen has located it in close proximity to *TERF1* [59]. *BLM* and *FEN1* both bind to *TERF2* and promote telomeric DNA synthesis via the ALT pathway [60,61,62,63]. They were both upregulated in EC. Despite being implicated in various cancer types, their role in EC has not been studied before [64,65,66,67,68]. Our methodology is validated by some TTAGPs relevant to EC that had previously been confirmed by other authors, for example; *FOXA1* was also a highly upregulated DEG, which is known to regulate oestrogen receptor binding in breast cancer [69]. It interacts with *NOP10* and *GAR1*—components of the telomerase complex [70]. A previous study has shown it to be overexpressed in EC compared to atypical hyperplasia and normal endometrium [71]. However, there is conflicting evidence regarding its effect on EC proliferation, with some studies proposing it stimulates growth, while others report an inhibitory effect [71,72,73]. Amongst the most significantly downregulated DEGs were *MYOCD, RSPO1, FOXL2* and *ARHGAP20*. This is also supported by the PCA, in which these genes were shown to contribute to separation of cancer samples and healthy controls. *FOXL2* is a telomerase TF and, consistent with our findings, a previous in vitro study has also reported *FOXL2* to have lower expression in EC tissues than normal endometrium [32,74]. Some of the newly identified DEGs have not previously been examined in EC, but possess confirmed pro-carcinogenic functionalities that could explain their observed changes in this pathology. *MYOCD, RSPO1* and *ARHGAP20* are examples of this. *MYOCD*, which encodes myocardin, is required for cardiac and smooth muscle development and is a potent transcriptional co-activator which acts in concert with telomerase [32,75,76]. *RSPO1* is involved in embryonic development and organogenesis and is predicted to interact with *hTERT* [77,78]. *ARHGAP20* contributes to cellular regulation processes and has been found within a protein network surrounding *TERF1*, *TERF2* and *POT1* [79,80]. *MYOCD, RSPO1* and *ARHGAP20* have all been implicated in various cancers, including lung cancer [75,77,79]. Along with *JSRP1, FEN1* and *BLM*, they have not been previously studied in EC and further investigation is warranted to understand how they may contribute to EC carcinogenesis.

The comparison of DEGs from different histological subtypes revealed that many genes were consistently dysregulated, compared with healthy tissue. *BLM, AURKA* and *PITX1* were upregulated in each subtype and were more significantly upregulated in carcinosarcoma tumours than endometrioid. AURKA is known to enhance telomerase activity by binding to TERF1 [81], whilst PITX1 suppresses hTERT transcription by binding to the *hTERT* promoter [47,82]. Carcinosarcoma is a highly aggressive subtype of EC, with patients typically exhibiting early metastasis, rapid disease progression and poor survival [83]. Consequently, greater upregulation of a gene in carcinosarcoma tumours may signify an association with more aggressive disease/poor prognosis. Overexpression of *BLM, AURKA* and *PITX1* has previously been linked with poor survival in breast, lung, bladder and pancreatic cancer [84,85,86,87,88]. Furthermore, *AURKA* has been shown to reduce EC cell proliferation and invasion in vitro and was associated with poor prognosis from the TCGA dataset [89]. Taken together with our findings, this suggests that *BLM, AURKA* and *PITX1* may contribute to more aggressive disease. From this analysis, we also identified multiple subtype-specific DEGs. *S100P* was only found to be overexpressed in endometrioid tumours. It is predicted to affect telomere biology due to its close proximity to *RAP1* [90]. Previous studies have also linked *S100P* expression with the squamous and adenosquamous subtypes of EC [91], but its association with endometrioid tumours has not been investigated. *H19*, which suppresses telomerase activity [92], was found to be highly downregulated in only the endometrioid subtype, in agreement with a previous study [93]. *IQSEC3* was also significantly downregulated and is predicted to affect telomere maintenance due to its telomeric location [94]. *XAGE2* and *PCP4* were serous-specific DEGs that are both thought to interact with *POT1* [90]. *IQSEC3, XAGE2* and *PCP4* have not been studied in EC previously, and further studies are necessary to investigate their associations with endometrioid and serous tumours. *MYOG*, which encodes myogenin, was only upregulated in carcinosarcoma tumours. Myogenin is a TF known to regulate myogenesis, and has also been shown to silence the *hTERT* gene [95]. It has not previously been studied in EC but has been linked with multiple sarcomatous cancers, such as rhabdomyosarcoma and leiomyosarcoma [96,97,98]. It may be a potential biomarker of carcinosarcoma tumours. From our analysis, we have identified many genes that may provide further insight into the pathogenesis of each of the subtypes and act as potential diagnostic/prognostic biomarkers or type specific molecular pathways. Many of these, such as *BLM, PITX1* and *MYOG*, have not been studied in EC previously and provide the basis for future experiments.

Genes dysregulated according to tumour grade included *CDC45* and *RAD51AP1*, which are both associated with the ALT pathway [99,100,101]. They have been linked with increased growth and progression in various cancers, including colorectal, ovarian and lung cancer [102,103,104], but have not previously been studied in EC. Between stages I and IV, *MAGEA4* and *TDRD10* were amongst the most highly upregulated DEGs, and *DUT* was the most downregulated. *MAGEA4* has been shown to be located in close proximity to *POT1* in a fluorescence localisation screen [90]. Previous studies have also linked overexpression of *MAGEA4* with the carcinosarcoma subtype and with poor survival in high grade EC [105,106]. *TDRD10* and *DUT* expression have both been linked with poor survival in cancer [107,108,109], but have never previously been studied in EC. *TDRD10* is predicted to be associated with telomere maintenance due to its role in DNA repair [100], whilst *DUT* has been found at the telomeres of telomerase and ALT positive cell lines [110]. *KIF4A*, which has been found at telomeres in a telomerase-positive cell line [110], was downregulated in both stage IV EC and grade 3 endometrioid cancer. In accordance with this, previous studies have shown that inhibition of *KIF4A* contributes to decreased EC cell proliferation in vitro [111]. By investigating differences in gene expression between the extremes of clinical stages and histological grades, many novel genes have been identified, such as *CDC45* and *RAD51AP1*, and their expression may indicate poor prognosis and play a role in the aggressiveness of the cancer.

Functional and pathway enrichment analysis of DEGs between EC and normal tissues revealed an association of those with DNA replication, cell cycle and regulation of transcription in EC. This is consistent with the proposed dysregulation of the cell cycle due to telomere-induced senescence in EC [12], enabling the EC cells to adapt their hallmark features such as replicative immortality [112]. Furthermore, many of these genes may further contribute to cellular immortality via their extra-telomeric functions in cell replication and tumour survival [113].

By constructing a PPI network, a significant module that had a functional role in DNA replication and cell cycle regulation was identified. From the network, most of the top 10 hub genes identified (*CDK1, CCNA2, CCNB1, PLK1, CDC6* and *AURKA*) were also associated with similar cell cycle regulatory functions [89,114,115,116,117]. This further reinforces the fundamental involvement of TTAGPs in cellular division. Our findings are further validated by the identification of hub genes *CCNA2, CDK1, AURKA* and *CCNB1* in the network of DEGs between EC and normal tissue, which are consistent with previous studies [118,119,120,121,122]. Therefore, it is not surprising that many of the identified hub genes have already been implicated in EC. Inhibition of *EZH2, CDK1, PLK1* and *AURKA* have been shown to suppress EC cell proliferation and invasion, and increase cellular apoptosis in vitro [89,123,124,125,126,127,128,129]. *PLK1*, *CDK1* and *AURKA* are involved in the phosphorylation of *TERF1*, which enables it to bind to telomeres as part of the shelterin complex [81,130]. Furthermore, a previous study has reported that *EZH2* overexpression may correlate with poor prognosis in EC, but this was not found in the TCGA dataset [127]. *EZH2* has been reported to interact with *TERF2* and *TERF2IP* [131], of the shelterin complex, and also telomeric repeat-containing RNA (*TERRA*) [132,133]. In our survival analysis, *AURKA* and *CCNA2* were identified as markers of unfavourable prognosis (Table S34), which is supported by previous immunohistochemical studies [122,134]. Polymorphisms within the *RAD51* gene have been associated with EC progression and recurrence [135,136]. *RAD51* is involved in homologous recombination repair, which is used to repair double strand breaks [137], and has been suggested to be part of the ALT pathway that utilises this repair mechanism to synthesise telomeric DNA [138,139]. The expression of *CCNA2, CCNB1*, *CDC6* and *GAPDH* have all been implicated in various cancers, including lung, ovarian and pancreatic cancer [140,141,142,143,144,145,146,147,148,149,150,151,152]. *CDC6* interacts with *TERF1* and increased expression is associated with upregulation of *hTERT* [153,154]. *CCNB1* expression has been shown to correlate with telomerase activity and *CCNA2* has been found at telomeres in an ALT-positive cell line [101,110]. *GAPDH* binds telomeric DNA and protects telomeres against rapid degradation in response to ceramide and chemotherapeutic agents [155,156,157]. The top hub genes amongst the DEGs between stage I and IV EC were *NOP56* and *NHP2,* which are both associated with poor prognosis in EC from the TCGA dataset [158]. NHP2 is a component of the telomerase complex (Figure 1) whilst NOP56 interacts with multiple components of the complex (Figure S3). It interacts with DKC1 and NOP10 and is predicted to bind NHP2 [159,160,161]. Many of the hub genes we identified appear to contribute to growth and progression of EC. *CDC6, CCNB1, GAPDH, NHP2* and *NOP56* are linked with carcinogenesis but have not been investigated previously in EC. Further studies are necessary to elucidate how they may contribute to EC pathogenesis.

Three enriched TFs were identified from the analysis of DEGs in EC and all were associated with telomere maintenance. E2F1 and MZF1 are both associated with downregulation of hTERT transcription and diminished telomerase activity, whereas ZEB1 upregulates hTERT expression [162,163,164,165,166,167]. E2F1 is involved in cell cycle regulation and apoptosis [168,169]. It regulates many cell cycle effector proteins such as CDC6 and CCNA2 [170,171]. It is upregulated in EC and associated with poor prognosis [169,172,173]. The upregulation of *E2F1* in EC is largely consistent with the expression of several of its target genes, such as *PDK4*, *BRCA1* and *FOXM1* [174,175,176], in our differential expression analysis. ZEB1 (zinc-finger E-box binding protein 1), which is known to promote epithelial-to-mesenchymal transition (EMT) [177,178], is associated with increased invasion and metastasis in EC [165,179,180,181,182,183,184]. *ZEB1* was downregulated in EC compared to healthy endometrium and the expression of its target genes, *RPS6KA5*, *DNMT3B*, *EPCAM* and *KLF4*, were generally consistent with this [185,186,187]. MZF1 is a SCAN domain-containing zinc finger protein which regulates transcription during various developmental processes [188]. Aberrant expression of MZF1 has been implicated in various cancer types, and can increase cancer cell proliferation, invasion and metastasis [166,188]. However, its role in EC has not been studied previously and remains to be clarified.

Multiple drugs already used in EC management were shown to interact with the identified hub genes and TFs; these included chemotherapeutic agents such as paclitaxel, carboplatin and doxorubicin [189]. In addition to this, our work highlighted metformin and mTOR inhibitors, such as everolimus, and they have already shown promise in early clinical trials for the treatment of EC [190,191,192,193,194,195]. In vitro studies have demonstrated the therapeutic benefit of AURKA inhibitors, cordycepin, genistein, suramin, sodium butyrate and ibrutinib [89,196,197,198,199,200]. The MEK inhibitor selumetinib has shown anti-tumour effects in EC cell culture [201], whilst binimetinib is yet to be studied in EC. The chemotherapy agents chlorambucil, carmustine and bendamustine are frequently used in the treatment of haematological cancers, such as non-Hodgkin lymphoma and chronic lymphocytic leukaemia, but are yet to be studied in EC [202,203,204,205]. Our data also identified many novel drug agents that demonstrate anti-tumour activity in vitro and in vivo, and these include the anti-mesothelin immunotoxin SS1 (dsFv)-PE38, the PI3K inhibitor AZD-6482 and the cyclin-dependent kinase inhibitors variolin B, meriolin, alsterpaullone and dinaciclib [206,207,208,209,210,211,212,213]. The therapeutic benefit of many of these drugs has not been investigated in EC and considering that they target key regulatory genes and TFs, it would seem prudent to assess their effectiveness in EC management.

The survival analysis revealed *ERBB2*, also known as *HER2*, to have the most significant association with poor prognosis in EC, in agreement with previous studies [214,215,216,217]. *ERBB2* stimulates *hTERT* promoter activity and increases *hTERT* transcription [218,219]. Other genes significantly associated with unfavourable prognosis included *ARL4C*, *TUBB4A* and *TPX2*. Previous immunohistochemical studies have found similar results for *ARL4C* and *TPX2* in EC [220,221], whereas no reports exist to date on *TUBB4A* in the endometrium. *ARL4C* has been shown to interact with *TERF2* and *TERF2IP* [222], whilst *TUBB4A* interacts with *TINF2* [160]. Knockdown of *TPX2* has been demonstrated to result in diminished telomerase activity and its overexpression has been linked with increased invasion and metastasis [45,223,224]. In accordance with this, it was also found to be upregulated in grade 3 endometrioid cancer. The genes most significantly associated with favourable prognosis included *ESR1, ANAPC4, RPS6KA1* and *WFS1*. *ESR1* is a telomerase activating factor that binds to the *hTERT* promoter and its role in EC is well established [47,217,225]. The identification of *RPS6KA1* and *WFS1* is interesting as previous studies have reported their role in the promotion of tumour progression and metastasis in various cancers [226,227,228,229]. The role of *ANAPC4* in cancer has not been studied in detail. *RPS6KA1* interacts with *TERF2IP* to mediate telomere shortening and *WFS1* had been found in close proximity to *TERF1* in a fluorescence localisation screen [90,230]. *ANAPC4* is predicted to influence telomere maintenance due to a yeast homologue having a role in telomere biology [231]. *CIRBP*, which has previously been found at the telomeres of a telomerase-positive cell line [110], was downregulated in stage IV disease and associated with a favourable prognosis. Previous studies have also linked loss of *CIRBP* expression with malignant progression of nasopharyngeal carcinoma [232]. *RPS6KA1*, *WFS1*, *ANAPC4* and *TUBB4A* have not been investigated in EC prior to this, and further studies are indicated to elucidate how these genes may affect survival in cancer in general, as well as their role in EC.

The limitations to this study are reflected by the well-known deficiencies in the TCGA dataset. For example, it does not include all different subtypes of EC, such as clear cell carcinomas, which constitute 2–3% of EC diagnoses, and is more frequently diagnosed than carcinosarcoma [233]. The pathogenesis of clear cell carcinomas is not well described and identifying dysregulated genes within this subtype may further our understanding [234]. Furthermore, the TCGA-UCEC dataset does not contain survival data for all patients included in this study, thus limits our survival analysis, and it is not completely representative of the carcinosarcoma patients included in the differential expression analysis. In addition, the survival analysis only considered the prognostic value of dysregulated genes in EC compared with healthy endometrium. There may be genes that are not aberrantly expressed in this comparison, but their expression in cancer may correlate with survival. An example of this is NOP56, which interacts with DKC1 and NHP2 in the telomerase complex (Figure S3) and is associated with poor prognosis in the TCGA dataset [158]. Finally, many of the genes and proteins identified are suspected to contribute to carcinogenesis via their roles in telomere biology in addition to other extra-telomeric functions. Alterations in telomere biology function of these TTAGPs are not likely to be their only causative involvement in endometrial carcinogenesis, but they are likely to be influencing the carcinogenic aberrations in various other important cellular functions such as cell cycle progression, transcription or DNA replication. The intricate relationship between telomere/telomerase biology with these essential cellular functions makes it impractical to completely disentangle the exact functional pathway(s) through which these multi-function TTAGPs contribute to endometrial carcinogenesis.

In summary, our study fills a void in the current literature with no prior in silico study investigating the relationship between dysregulated or prognostic genes in EC relevant to telomerase and telomere maintenance. This study has highlighted that telomere maintenance underpins the functions of many of these genes and provides a novel outlook on EC pathogenesis and prognosis. Through our in silico methods, we have identified many critical genes associated with telomere maintenance, which are previously unknown to contribute to endometrial carcinogenesis and prognosis, such as *NOP56*, *WFS1*, *ANAPC4* and *TUBB4A*. Further studies in a local, prospective cohort are required to validate these in silico results. Many of the potential biomarkers we have identified not only provide avenues for further research in EC, but our methods and protocol can be used as a template for initial hypothesis generating study into the role of TTAGPs in other cancers.

## Figures and Tables

**Table 1:** Transcription factors (TFs) whose binding sites were enriched in the DEGs, and their associated Fisher score. oPOSSUM software was used to identify TFs with a Fisher score greater than 2 standard deviations above the mean.

TF	Fisher Score
E2F1	49.853
MZF1_5-13	54.086
ZEB1	50.209

## Supplementary Materials

The following are available online at https://www.mdpi.com/2409-9279/3/3/63/s1, Figure S1: TFBS Z-Score and Fisher Score, Figure S2: PCA, Figure S3: Telomerase and Shelterin Complex Interactions, Table S1: Telomere and Telomerase Associated Genes and Proteins, Table S2: TCGA RNASeqV2 Normalised Expression Data, Table S3: TCGA-UCEC Clinical Data, Table S4: TCGA-UCS Clinical Data, Table S5: DEGs-Cancer-Healthy, Table S6: DEGs-Endometrioid-Healthy, Table S7: DEGs-Serous-Healthy, Table S8: DEGs-Carcinosarcoma-Healthy, Table S9: Common Upregulated Genes Between EC Subtypes, Table S10: Common Downregulated Genes Between EC Subtypes, Table S11: DEGs-Endometrioid Grade 3–1, Table S12: DEGs-Stage IV-I, Table S13: Biological Process GO Terms for DEGs (Enrichr), Table S14: Molecular Function GO Terms for DEGs (Enrichr), Table S15: Cellular Compon ent GO Terms for DEGs (Enrichr), Table S16: KEGG Pathways for DEGs (Enrichr), Table S17: Revised Biological Process GO Terms for DEGs (REVIGO), Table S18: Revised Molecular Function GO Terms for DEGs (REVIGO), Table S19: Revised Cellular Component GO Terms for DEGs (REVIGO), Table S20: PPI Network Nodes, Table S21: Nodes of MCODE Module, Table S22: Biological Process GO Terms for Module (Enrichr), Table S23: Molecular Function GO Terms for Module (Enrichr), Table S24: Cellular Component GO Terms for Module (Enrichr), Table S25: KEGG Pathways for Module (Enrichr), Table S26: Revised Biological Process GO Terms for Module (REVIGO), Table S27: Revised Molecular Function GO Terms for Module (REVIGO), Table S28: Revised Cellular Component GO Terms for Module (REVIGO), Table S29: Stage IV-I DEGs–Node Scores, Table S30: TFBS, Table S31: TFBS Control Analysis 1, Table S32: TFBS Control Analysis 2, Table S33: Therapeutic Targets, Table S34: Survival Analysis, Table S35: Common DEGs Between Grades, Stages and Prognosis.
