# Supplementary material for: Investigating the Role of Telomere and Telomerase Associated Genes and Proteins in Endometrial Cancer
Source: Methods Protoc. 2020 Sep 3;3(3):63. doi: 10.3390/mps3030063 (PMC7565490; doi:10.3390/mps3030063)
Supplement: Supplementary file 1 [file mps-03-00063-s001.zip › Supplementaries/Figure S2. PCA.pdf]

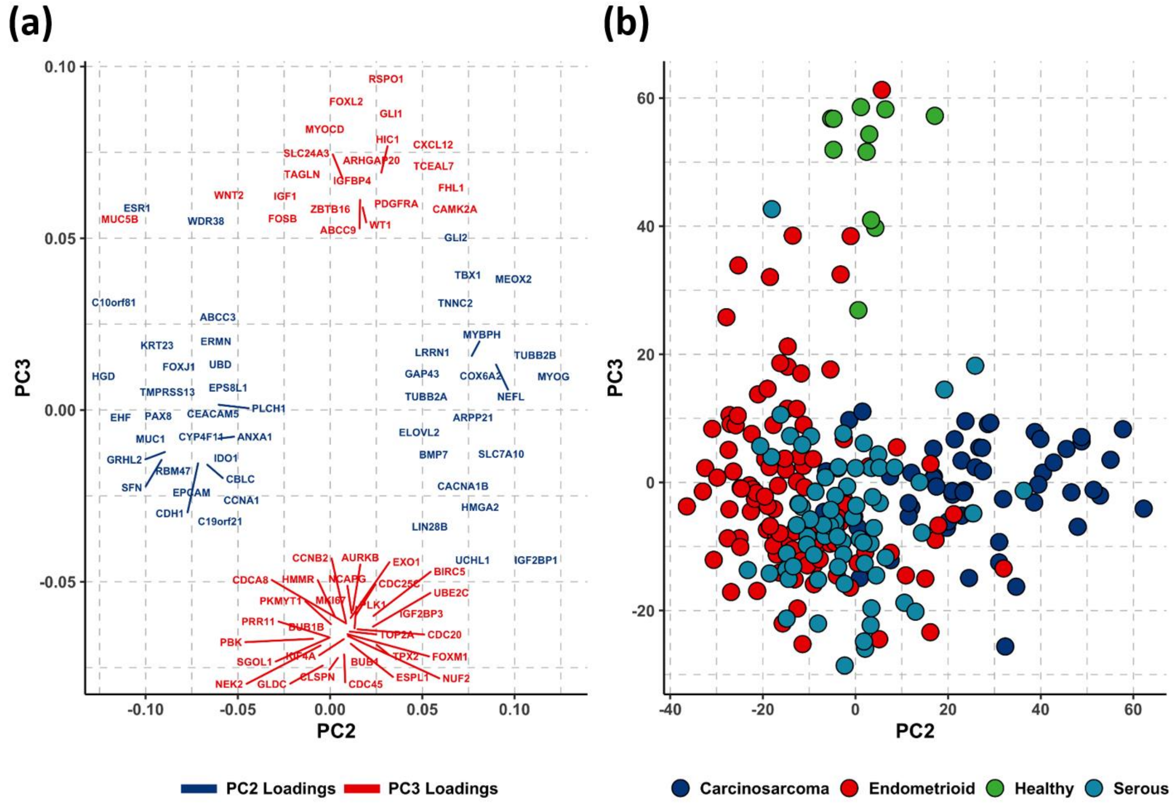

Figure S2. (**a**) Loading plot of the top 50 genes contributing to variance from principal components (PCs) 2 and 3. (**b**) Principal component analysis (PCA) of log normalised counts per million (CPM) expression data displaying PC2 (10.45% variance) and PC3 (6.95% variance) with samples coloured by cancer subtype.
