# Supplementary material for: Investigating the Role of Telomere and Telomerase Associated Genes and Proteins in Endometrial Cancer
Source: Methods Protoc. 2020 Sep 3;3(3):63. doi: 10.3390/mps3030063 (PMC7565490; doi:10.3390/mps3030063)
Supplement: Supplementary file 1 [file mps-03-00063-s001.zip › Supplementaries/Figure S3. Telomerase and Shelterin Complex Interactions.pdf]

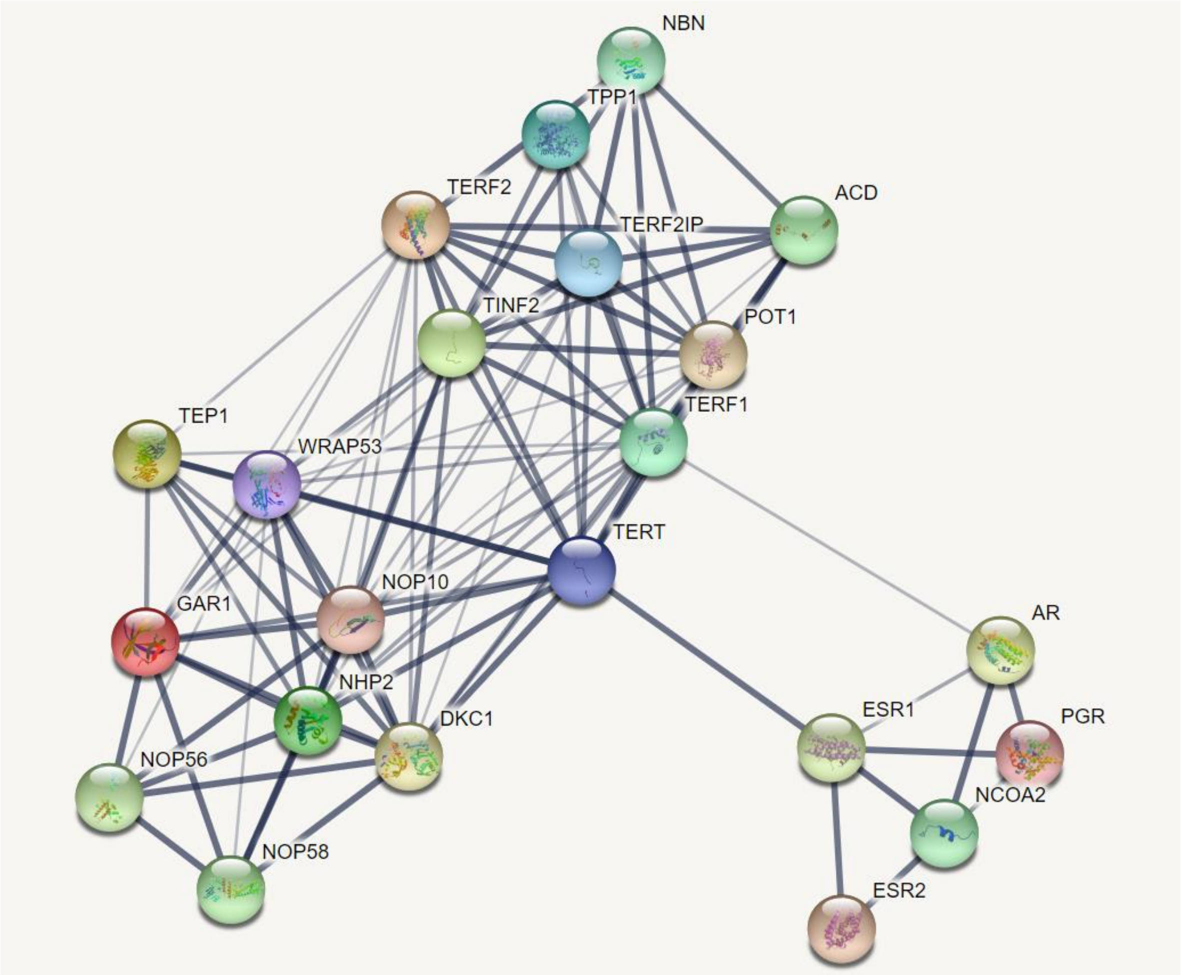

Figure S3. Telomerase and Shelterin Complex Interactions. Diagram demonstrating the interactions between the components of the telomerase and shelterin complexes, and steroid hormone receptors expanded by one level in STRING.
