# Supplementary material for: Investigating the Role of Telomere and Telomerase Associated Genes and Proteins in Endometrial Cancer
Source: Methods Protoc. 2020 Sep 3;3(3):63. doi: 10.3390/mps3030063 (PMC7565490; doi:10.3390/mps3030063)
Supplement: Supplementary file 1 [file Figure S1. TFBS Z-score and Fisher Score.pdf › Supplementaries/Figure S1. TFBS Z-score and Fisher Score.pdf]

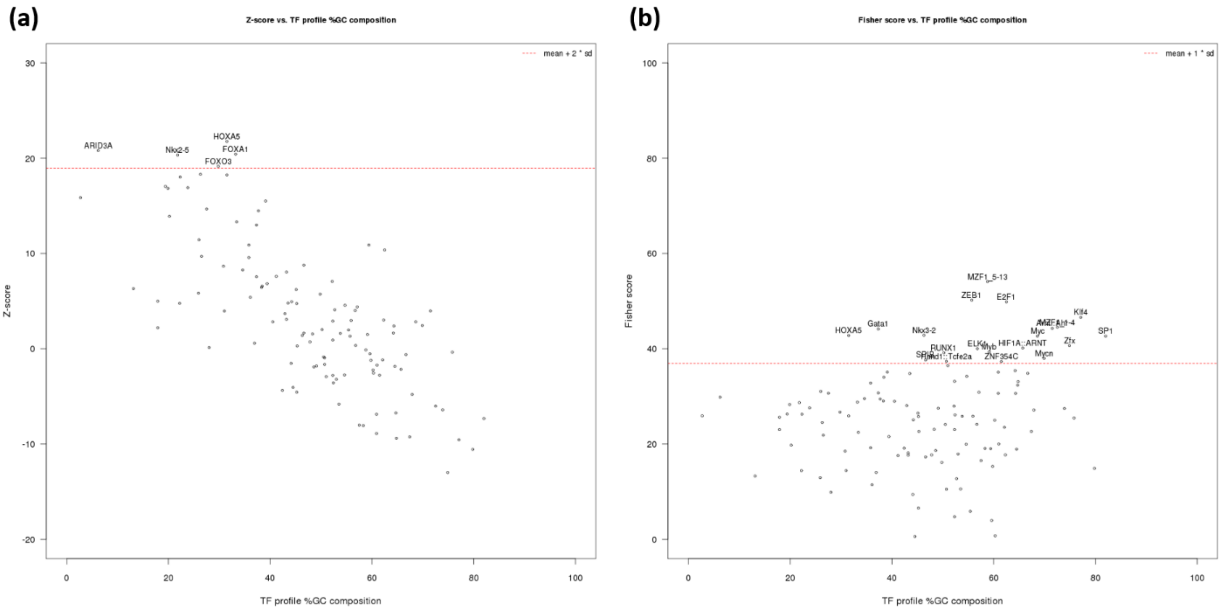

Figure S1. Transcription factors (TF) whose binding sites were enriched in DEGs, analysed using oPOSSUM software. (**a**) Graph displaying Z-score against the GC composition of TFs, and the bias for the TF with a lower GC composition to have a higher Z-score. (**b**) Graph displaying Fisher score against the GC composition of TFs. TFs have a more even distribution and do not show bias towards a low or high GC composition.
